# Metal ion scavenging activity of elastin-like peptide analogues containing a cadmium ion binding sequence

**DOI:** 10.1038/s41598-022-05695-w

**Published:** 2022-02-03

**Authors:** Shogo Sumiyoshi, Keitaro Suyama, Daiki Tatsubo, Naoki Tanaka, Keisuke Tomohara, Suguru Taniguchi, Iori Maeda, Takeru Nose

**Affiliations:** 1 Laboratory of Biomolecular Chemistry, Department of Chemistry, Faculty and Graduate School of Science, Kyushu University, Fukuoka, 819-0395 Japan; 2 Laboratory of Biomolecular Chemistry, Faculty of Arts and Science, Kyushu University, Fukuoka, 819-0395 Japan; 3 Department of Physics and Information Technology, Kyushu Institute of Technology, Iizuka, Fukuoka 820-8502 Japan

**Keywords:** Peptides, Environmental chemistry

## Abstract

The development of simple and safe methods for recovering environmental pollutants, such as heavy metals, is needed for sustainable environmental management. Short elastin-like peptide (ELP) analogues conjugated with metal chelating agents are considered to be useful as metal sequestering agents as they are readily produced, environment friendly, and the metal binding domain can be selected based on any target metal of interest. Due to the temperature dependent self-assembly of ELP, the peptide-based sequestering agents can be transformed from the solution state into the particles that chelate metal ions, which can then be collected as precipitates. In this study, we developed a peptide-based sequestering agent, AADAAC-(FPGVG)_4_, by introducing the metal-binding sequence AADAAC on the *N*-terminus of a short ELP, (FPGVG)_4_. In turbidity measurements, AADAAC-(FPGVG)_4_ revealed strong self-assembling ability in the presence of metal ions such as Cd^2+^ and Zn^2+^. The results from colorimetric analysis indicated that AADAAC-(FPGVG)_4_ could capture Cd^2+^ and Zn^2+^. Furthermore, AADAAC-(FPGVG)_4_ that bound to metal ions could be readily recycled by treatment with acidic solution without compromising its metal binding affinity. The present study indicates that the fusion of the metal-binding sequence and ELP is a useful and powerful strategy to develop cost-effective heavy metal scavenging agents with low environmental impacts.

## Introduction

In the recent years, contamination of toxic heavy metal ions in industrial wastewater has become a major environmental concern in many parts of the world^1, 2^. While some heavy metal pollutants are metabolized and decomposed chemically or biologically by organisms, others tend to get accumulated in the body. Especially, cadmium and cadmium-containing compounds are known to be carcinogens that induce many types of cancer and neurological diseases such as itai–itai disease^3^. Therefore, effective remediation methods to remove cadmium ions from soil and water are of increasing interest and importance. To date, diverse treatment strategies for cadmium contaminants, including chemical precipitation, membrane separation, conventional coagulation, ion exchange, reverse osmosis, and polymer or carbon nanocomposite absorbents, have been explored^3–9^. Among them, ion exchange and activated carbon absorbents have been used as general procedures for removing cadmium ions^10, 11^. However, the use of these methods is limited due to high cost and tedious regeneration of adsorbents. Thus, the development of highly efficient and low-cost methods to remove cadmium are urgently required. In addition, there is also an increasing demand for developing environment friendly cadmium removal methods as compared to the existing harsh chemical methods.

Biomaterials composed of natural materials are attracting attention as they have small environmental impact. Thermoresponsive biomaterials can modify their structures and properties in response to changes in temperature^12^. Elastin-like peptides (ELPs) are representative thermoresponsive biomaterials whose properties have been widely explored. ELPs are artificial peptides derived from elastin, which is a core protein of the elastic fibers in connective tissues^13^. These peptides exhibit temperature-dependent reversible phase transition known as “coacervation” with lower critical solution temperature (LCST) behavior under physiological conditions. These materials have attracted the attention of researchers owing to their various potential applications as drug-delivery systems (DDS)^14–18^, protein separation supports^19–22^, and metal scavenging agents^23–29^. Owing to their thermoresponsive properties, ELPs are soluble in water below a transition temperature (*T*_t_) and are insoluble above *T*_t_^30^. Therefore, ELP analogues fused with metal binding domains can be used as adsorbents to remove or detect heavy metals in aqueous solution over temperature changes. Owing to their ease of production, biocompatibility, biodegradability, and selective tailoring of the metal binding domain toward any target metal of interest, ELP-metal binding domain fusion copolymers are expected to work as an effective absorbent for heavy metals.

Temperature sensitivity of ELPs is an important factor in the development of thermoresponsive biomaterials. It has been envisaged that the characteristic repetitive sequences of ELPs are important for this sensitivity^13^. Elastin possesses several hydrophobic repetitive sequences comprising consecutive 3–6 amino acid residues^13^. Among the different sequences, the pentapeptide sequence Val-Pro-Gly-Val-Gly (VPGVG) is the most commonly identified repeating motif in vertebrate species^31–33^, and apparently exhibits coacervation due to the temperature sensitivity of the sequence^34–36^. Therefore, the (VPGVG)_n_ polypeptide has been studied for its temperature-responsive behavior and structural features as one of the benchmarks of ELPs. Several factors, such as the amino acid sequence, the number of hydrophobic amino acids present, the molecular weight (i.e., the number of peptide repeats), the concentration of ELP, the pH of the solution, and ionic strength, are known to influence the coacervation of ELPs ^37–40^. The amino acid sequence of ELPs has a marked effect on the coacervation ability^39, 41^. Previously, it was reported that substitution of residue X in the XPGVG repetitive sequence with other amino acids caused strong aggregation and altered the *T*_t_ of the ELPs^42, 43^. We found that phenylalanine-containing synthetic ELPs consisting of (FPGVG)_n_ sequences exhibited strong coacervation ability at a significantly low number of repetitions (n = 5)^44^, whereas (VPGVG)_n_ required a relatively high repetition number (n > 40) to exhibit coacervation ability^45–47^. Therefore, by mimicking such repetitive sequences, various ELP analogues composed of Xaa-Pro-Gly-Val-Gly (XPGVG where X = I, F, and W) pentapeptide repeats have been developed^44, 47–51^. During these experimental studies, we discovered that nitrilotriacetic acid (NTA)-conjugated short ELP analogues could bind to several metal ions, such as copper or nickel^52^. This result indicated that short H-(FPGVG)_n_-NH_2_ analogues (n = 3–5), henceforth abbreviated as Fn, could be used as the temperature-responsive component of ELP-based metal scavengers. To date, ELP-based metal scavengers have been developed by using relatively long elastin-derived polypeptides with a molecular weight of approximately 40–50 kDa^27–29^. Since short ELPs, such as Fn (n = 3–5), can be prepared by fragment condensation, they can be chemically synthesized at a lower cost than the long-chain peptides, which require an *Escherichia coli* expression system, Fn analogues fused with metal-binding domains can be utilized as low-cost and environmentally friendly metal scavengers.

For application of ELP analogues as metal scavenging agents, it is important to adjust the metal-binding functionalities fused with ELPs to improve their affinity and specificity for desired heavy metal ions. One of the strategies for producing peptide-based metal scavenging agents is to fuse metal-binding peptide sequences with temperature-responsive peptides such as ELPs^52^. Won et al. generated phytochelatin–ELP fusion proteins with temperature sensitivity and metal-binding functionality to remove heavy metal ions biologically^28, 29^. In another report, Kostal et al*.* proposed the synthesis of polyhistidine–ELP fusion proteins that can remove heavy metals from dilute waste streams^23^. In the current study, the Ala-Ala-Asp-Ala-Ala-Cys (AADAAC) sequence reported by Lihi et al*.*, was selected as a metal-binding functional group, which binds to heavy metal ions such as cadmium^53^. This hexapeptide sequence strongly and selectively binds to cadmium under slightly acidic and neutral pH conditions. In addition, this peptide releases metal ions under highly acidic conditions (pH < 4.0). Owing to these characteristics, we hypothesized that AADAAC-ELP could be readily regenerated by a simple acid treatment after using it as a metal scavenger. Therefore, we attempted to design novel heavy metal scavenging agents by combining the AADAAC sequence with short Fn peptides possessing strong coacervation activity. These agents can be easily produced, are environmentally friendly, exhibit high affinity for cadmium ions, and can desorb adsorbed metal ions.

We, herein, have synthesized a novel ELP analogue, AADAAC-F4, by introducing the metal-binding AADAAC sequence at the *N*-terminus of F4, and investigated its potential use as a cadmium removing agent. Synthesized peptides were evaluated for their coacervation activity using the turbidity measurement. To investigate the cadmium binding affinity of AADAAC-F4, colorimetric analysis using xylenol orange (XO) and inductively coupled plasma mass spectroscopy (ICP-MS) were carried out. Regeneration and reusability of AADAAC-F4, after using it as a cadmium absorbent, were assessed after a simple acid treatment. The temperature-dependent self-assembly of AADAAC-F4 was also confirmed by particle size distribution measurements. In addition, the morphology of AADAAC-F4 in the presence and absence of cadmium ions was investigated by optical and scanning electron microscopy. Finally, the binding dynamics of metal ions to the AADAAC-F4 peptide were investigated using calorimetric measurements. As a result, we have observed that AADAAC-F4 reveals strong self-assembling ability in the presence of Cd^2+^ ions and is also able to act as a Cd^2+^ ion scavenger.

## Results and discussion

### Synthesis and purification of peptides

**Figure 1:**
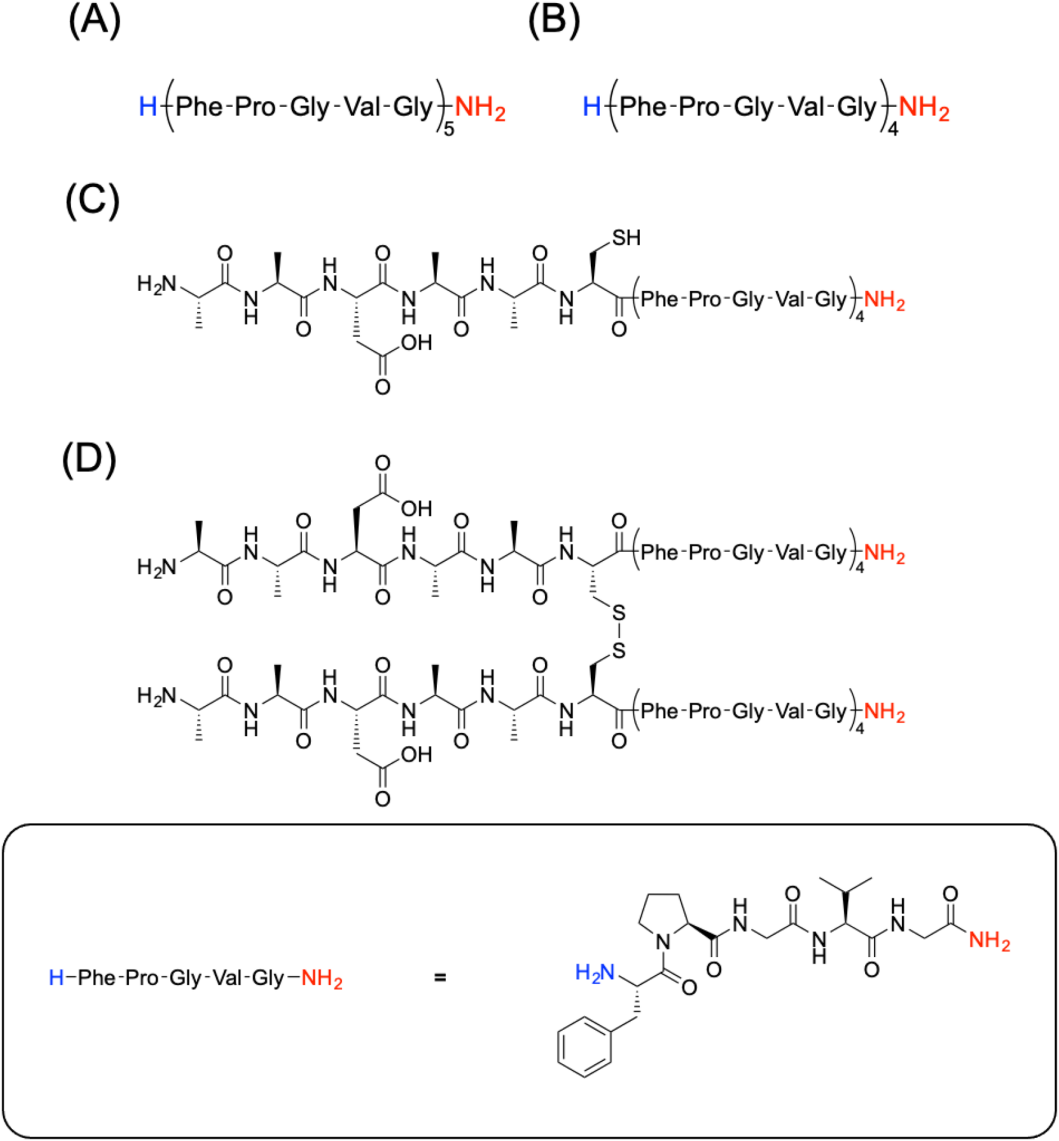
Chemical structures of the synthesized peptide analogues. The chemical structures of (**A**) F5, (**B**) F4, (**C**) AADAAC-F4, and (**D**) AADAAC-F4-dimer are shown. The *C*-terminus of each peptide was capped with an amide group to avoid side reactions (represented using red letters).

To obtain peptide-based sequestering agents, ELP analogues, namely F5, F4, and AADAAC-F4, were synthesized by the conventional solid phase peptide synthesis procedure. The chemical structures of the synthesized peptides are shown in Fig. 1. Synthesized peptides were purified by reversed-phase (RP)-HPLC. In addition, a dimerized analogue of AADAAC-F4 (AADAAC-F4-dimer) was synthesized by disulfide-bond formation between the cysteine residues. Previously, we reported that dimeric short Fn analogues dimerized with a disulfide bond exhibited strong coacervation activity^48^. Thus, it was hypothesized that the dimeric AADAAC-F4 analogue might be used as a metal scavenging agent at lower peptide concentrations. On the other hand, it was revealed that the thiol group of the Cys residue in the AADAAC sequence plays an important role in metal bonding^53^. Using the dimeric peptide AADAAC-F4, we investigated the molecular mechanism underlying the coacervation and metal binding potency of AADAAC-F4; especially the importance of the free-thiol group of the cysteine residue. The purity and molecular weight of each peptide were confirmed by RP-UPLC-MS (Table 1 and Fig. S1). The results indicated that the peptide analogues were obtained successfully with high purity.

**Table 1:** Fn analogues synthesized in this study.

Peptide	Yield (%)	Retention time (min)	MS (ESI) *m/z*		
			Composition formula	Calculated mass (u)	Found mass (u)
F5	36.8	2.496	C_115_H_158_N_26_O_25_	1153.10 [M + 2H]^2+^	1153.31
F4	42.8	2.251	C_92_H_127_N_21_O_20_	923.99 [M + 2H]^2+^	924.56
AADAAC-F4	28.3	2.350	C_111_H_157_N_27_O_28_S	784.06 [M + 3H]^3+^	784.39
AADAAC-F4-dimer	34.1^a^	2.992	C_222_H_312_N_54_O_56_S_2_	1175.08 [M + 4H]^4+^	1175.22

Retention times of each peptide were determined by RP-UPLC-MS.

^a^Yield when 50.64 mg of AADAAC-F4 was used as a raw material.

### Turbidity measurement of AADAAC-F4

**Figure 2:**
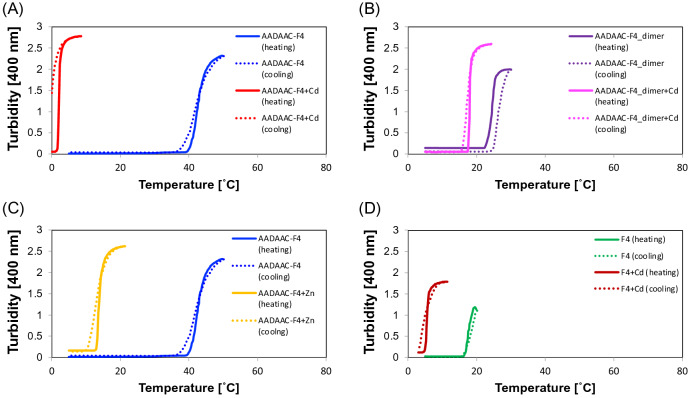
Turbidity profiles of AADAAC-F4, AADAAC-F4-dimer, and F4. Turbidity changes of the synthesized peptide in Tris–HCl buffer solution (50.0 mM Tris, 599 mM NaCl, pH 8.0) associated with heating (solid lines) and cooling (dashed lines). (**A**) AADAAC-F4, 4.26 mM (blue lines); and AADAAC-F4, 4.26 mM with 4.26 mM CdCl_2_ (red lines). (**B**) AADAAC-F4-dimer, 2.13 mM (purple lines); and AADAAC-F4, 2.13 mM with 4.26 mM CdCl_2_ (magenta lines). (**C**) AADAAC-F4, 4.26 mM (blue lines); and AADAAC-F4, 4.26 mM with 4.26 mM ZnCl_2_ (yellow lines). (**D**) F4, 5.42 mM (green lines); and F4, 5.42 mM with 4.26 mM CdCl_2_ (brown lines).

The synthesized peptide analogues were examined for their temperature-dependent self-aggregating activity. For the application of AADAAC-F4 as a metal scavenging agent, it was important to adjust the self-assembly ability of the peptide at which the peptide usually exhibits phase transition at a desired temperature and peptide concentration. Accordingly, turbidity measurements of AADAAC-F4 were conducted to determine its coacervation ability at 10 mg/mL in Tris–HCl buffer (pH 8.0) while varying the temperature. This concentration was used as a standard, and AADAAC-F4 dissolved well in aqueous solvents at this concentration. Measurements were conducted in the presence or absence of Cd^2+^ to investigate the effect of Cd^2+^ on the coacervation ability of the peptides (Fig. 2 and Table 2). The phase transition temperature (*T*_t_) was calculated from the change in turbidity for quantitative evaluation of the coacervation ability. When AADAAC-F4 was dissolved in Tris–HCl buffer, turbidity changes were not observed even when the solution temperature was elevated to 90 °C (Fig. S2). From previous findings, it is known that the coacervation ability of ELPs is markedly affected by the peptide sequence^37, 39, 41^. An increase in the ratio of hydrophobic residues in ELPs tends to decrease *T*_t_, while an increase in the ratio of hydrophilic residues tends to increase *T*_t_^37^. Before the measurement, AADAAC-F4 was considered to have a weak self-assembly ability due to the presence of hydrophilic Asp and Cys residues in the attached AADAAC sequence. Turbidity was measured in Tris–HCl buffer containing 3.5% NaCl, which is equivalent to seawater. Previously, salts such as NaCl have been reported to decrease the *T*_t_ of ELPs^54^. The addition of NaCl is useful for measuring peptide aggregation with high sensitivity while mimicking the environment in seawater, one of the places where the metal scavenging agents are frequently used. We observed that in the presence of salt, the *T*_t_ of AADAAC-F4 was 45.2 ± 0.9 °C in Cd^2+^-free solution. This result indicated that AADAAC-F4 could exhibit coacervation ability at a concentration of 10 mg/mL in a solution containing 3.5% NaCl and no Cd^2+^ ions. Remarkably, in the presence of Cd^2+^, AADAAC-F4 showed coacervation with an extremely low *T*_t_ value (2.2 ± 0.5 °C). This result suggested that the *T*_t_ of AADAAC-F4 was greatly affected by the Cd^2+^ present in the solution. It was observed that the turbidity in the Cd^2+^-containing solution did not return immediately to the initial value when the solution was re-cooled to 0 °C; further incubation (ca. 30 min) of the solution at 0 °C was required for reversion to a homogeneous solution. Thus, it was suggested that AADAAC-F4 formed stable coacervates in the presence of Cd^2+^. Previously, it has been reported that Cd^2+^ and the AADAAC sequence, in the ratio of 1:2, formed chelates^53^. When two molecules of AADAAC-F4 bound to one Cd^2+^ ion, peptide-metal complexes with a pseudo dimer-like structure were formed. Thus, the proximity of the peptide chains resulting from the chelation was hypothesized to lead to the improvement of self-assembling ability, similar to that observed for the F5-Cys-dimer^48^. To confirm this hypothesis, we evaluated the coacervation ability of the AADAAC-F4 dimer by adjusting the concentration of the F4 moiety to be the same as that of the monomer AADAAC-F4. It was revealed that the *T*_t_ value of the dimer was lower than that of the monomer in the Cd^2+^-free solution. However, contrary to the hypothesis, the *T*_t_ value of the dimer was higher than that of the monomer in the presence of Cd^2+^. This result indicated that the drastic decrease of *T*_t_ of AADAAC-F4 in the presence of Cd^2+^ was not simply due to the formation of the dimer-like structure consisting of two AADAAC-F4 moieties. Furthermore, the *T*_t_ of the AADAAC-F4 dimer was not strongly affected by the presence or absence of Cd^2+^ as compared to the monomer. This suggested that the free thiol group of the Cys residue of AADAAC-F4 is required for the enhancement of coacervation ability via binding to Cd^2+^. Turbidity measurements were also conducted in the solution containing Zn^2+^ as it has been reported that the AADAAC sequence also binds to Zn^2+^ ions^53^. It was observed that the *T*_t_ value of AADAAC-F4 was significantly affected by Zn^2+^, resulting in a decrease to 13.5 ± 0.1 °C in its presence. Accordingly, we concluded that the metal binding ability of AADAAC to Cd^2+^ and Zn^2+^ ions contributes to the enhanced self-assembly of AADAAC-F4 when coexisting with metal ions.

**Table 2:** The phase transition temperature (*T*_t_) values of the synthesized peptide analogues.

Peptide	Concentration		*T*_t_ (°C)
	mg/mL	mM	
AADAAC-F4	10	4.26	45.2 ± 0.9
AADAAC-F4 + Cd^2+^	10	4.26	2.2 ± 0.5
AADAAC-F4 + Zn^2+^	10	4.26	13.5 ± 0.1
AADAAC-F4-dimer	10	2.13	22.1 ± 1.0
AADAAC-F4-dimer + Cd^2+^	10	2.13	19.5 ± 0.8
F4	10	5.42	17.8 ± 0.1
F4 + Cd^2+^	10	5.42	6.8 ± 0.5
F5	5.0	2.17	13.7 ± 0.5

Mean *T*_t_ values with SE were shown in the table. Each peptide was dissolved in Tris–HCl buffer solution (50.0 mM Tris, 599 mM NaCl, pH 8.0) in the presence or absence of 4.26 mM CdCl_2_ or ZnCl_2_. The measurements were repeated at least three times.

As a control, F4, which has no metal binding domain, was evaluated for its coacervation activity. Since F4 does not contain the hydrophilic AADAAC sequence, it showed a lower *T*_t_ than that observed for AADAAC-F4 in the absence of Cd^2+^. We next measured the coacervation activity of F5, a previously reported representative ELP, for comparison. F5, an ELP analogue with a longer hydrophobic repeat than that in F4, showed coacervation at a lower *T*_t_ than that observed for F4 (F5: 13.7 °C and F4: 17.8 °C) (Fig. S3). It should be noted that the *T*_t_ of F4 was also moderately reduced by addition of Cd^2+^ (from 17.8 to 6.8 °C) (Table 2). Therefore, it was suggested that the interactions between F4 and Cd^2+^ solely enhanced the coacervation properties of this analogue. However, it should be considered that AADAAC-F4, AADAAC-F4-dimer, and F4 interact with Cd^2+^ in different ways. When metal ions are present in solution, AADAAC-F4 is known to coordinate with and bind to metal ions mainly through hydrophilic residues such as D and C residues on the AADAAC sequence^53^. This would greatly increase the hydrophobicity of this peptide and significantly decrease the *T*_t_. AADAAC-F4-dimer was able to interact with Cd^2+^, although it lacked the free thiol group; however, its effect on *T*_t_ was relatively very small compared to that of the monomer. In other words, AADAAC-F4-dimer may interact with Cd^2+^ via interactions other than those with the free thiol group. Similarly, F4, which consists of amino acids that do not have side chains with charges or hydroxyl groups, can also form interactions with metal ions, which can lower its *T*_t_. Overall, it was confirmed that AADAAC-F4 showed high water solubility in the metal-free solution and strong self-association ability in the presence of the metal ions. In addition, Fn (n = 4, 5) and AADAAC-F4-dimer also exhibited coacervation, although each peptide showed a different *T*_t_ value. Therefore, these temperature-dependent phase transition characteristics of AADAAC-F4 and other synthesized peptide analogues can render them suitable as reusable metal scavenging agents.

### Metal binding affinity of AADAAC-F4

**Figure 3:**
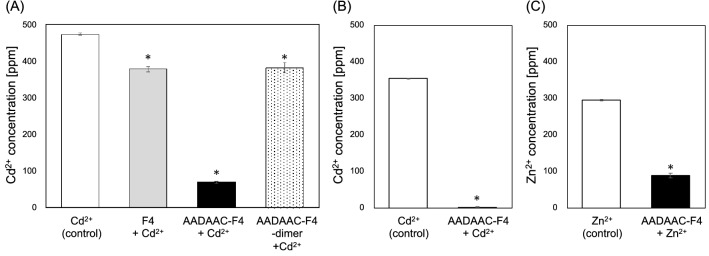
Metal binding affinity of AADAAC-F4 analogues. (**A**) Concentration of Cd^2+^ in the supernatant of the CdCl_2_ solution (4.26 mM) measured after treatment with F4, AADAAC-F4, and AADAAC-F4-dimer at a concentration of 10 mg/mL. The concentration ratio of AADAAC-F4 and Cd^2+^ was 1:1. (**B**) Concentration of Cd^2+^ in the supernatant of the CdCl_2_ solution (3.19 mM) measured after treatment with 10 mg/mL (4.26 mM) of AADAAC-F4. The concentration ratio of AADAAC-F4 and Cd^2+^ was 1.3:1. (**C**) Concentration of Zn^2+^ in the supernatant of the ZnCl_2_ solution (4.26 mM) measured after treatment with 10 mg/mL (4.26 mM) of AADAAC-F4. The concentration ratio of AADAAC-F4 and Zn^2+^ was 1:1. *P < 0.05 in a *t*-test between the concentration after treatment with each peptide compared to that of the control in each graph.

To investigate the binding affinity between AADAAC-F4 and metal ions, colorimetric analysis was carried out using the aqueous solution of CdCl_2_ and ZnCl_2_ (Fig. 3 and Table 3). F4 was used as a reference for the colorimetric analysis. The peptide and equivalent concentration of the metal ion were dissolved in 50 mM Tris–HCl (pH 8.0). The resulting solution was incubated at 40 °C for 1 h to separate the coacervates and equilibrium solution phases. Then, the concentration of metal ions that remained in the supernatant was detected by the colorimetry. As shown in Fig. 3A, when the peptide and Cd^2+^ were dissolved in a 1:1 ratio (both were 4.26 mM), the Cd^2+^ concentration in the supernatant was significantly reduced to 14.5% (the removal rate was 85.5%) upon the AADAAC-F4 treatment. This indicated that AADAAC-F4 molecules could potently capture cadmium ions in the aggregates formed by coacervation. In addition, it was revealed that the removal rate of Cd^2+^ changed linearly depending on the molar concentration of AADAAC-F4 (Fig. S4 and Table S1). When a small excess amount (1.3-fold) of AADAAC-F4 (10 mg/mL, 4.26 mM) was used to bind Cd^2+^ (3.19 mM, metal: peptide = 1: 1.3 in a ratio), the Cd^2+^ was almost completely removed from the supernatant (Fig. 3B). This result indicated that AADAAC-F4 can potentially be applied as a strong Cd^2+^ scavenger. On the other hand, when the Cd^2+^ solution was treated with F4, which did not have the AADAAC sequence, the Cd^2+^ concentration in the supernatant was reduced to 79.1%; in other words, the removal rate was only 20.9%. Since this partial reduction in the Cd^2+^ concentration could be attributed to metal adsorption on the F4 peptides themselves, the strong binding of AADAAC-F4 to Cd^2+^ was inferred to be due to the adsorption of metal by the AADAAC sequence. Subsequently, the binding affinity of AADAAC-F4-dimer to Cd^2+^ was also determined. The reduction of Cd^2+^ concentration by AADAAC-F4-dimer was almost identical to that of F4. Although the Cd^2+^ removal rate by the AADAAC-F4-dimer also changed linearly depending on the molar concentration, the Cd^2+^ removal rate by the dimer was only 31.5%, even when an equimolar amount of the peptide was used with Cd^2+^ (Fig. S4 and Table S1). Subsequently, it was confirmed that the unmodified AADAAC sequence, which possesses a free thiol group, is necessary for a favorable metal binding ability. In addition, to maintain the high metal interaction ability of AADAAC-F4, it is necessary to store and use AADAAC-F4 in a condition that prevents it from forming dimers through disulfide bonding.

**Table 3:** The removal rate of metal ions with the peptide treatment.

	Concentration of peptides		Concentration of metal ion	Removal rate
	mg/mL	mM	ppm	%
F4: Cd^2+^ (ratio = 1:1)	0	0	477.9 ± 5.6	20.9 ± 0.01
	10	5.42	378.2 ± 0.5	
AADAAC-F4: Cd^2+^ (ratio = 1:1)	0	0	490.5 ± 2.6	85.5 ± 0.01
	10	4.26	69.7 ± 3.4	
AADAAC-F4: Cd^2+^ (ratio = 1.3:1)*	0	0	354.6 ± 0.01	99.5 ± 0.002
	10	4.26	1.8 ± 0.5	
AADAAC-F4-dimer: Cd^2+^ (ratio = 1:2)	0	0	477.9 ± 5.6	20.1 ± 0.03
	10	2.13	382.0 ± 14.3	
AADAAC-F4: Zn^2+^ (ratio = 1:1)	0	0	295.2 ± 0.01	69.9 ± 0.02
	10	4.26	88.9 ± 5.9	

Mean removal rate with SE were shown in the table. Each peptide was dissolved in Tris–HCl buffer solution (50.0 mM Tris, 599 mM NaCl, pH 8.0). The measurements were repeated at least three times.

*Small excess amount (1.3-fold) of AADAAC-F4 (10 mg/mL, 4.26 mM) was used to Cd^2+^ (3.19 mM, metal: peptide = 1: 1.3 in a ratio).

Similar measurements were also performed for zinc ions (Zn^2+^) using an aqueous solution of ZnCl_2_ in the presence of AADAAC-F4. As shown in Fig. 3C, the Zn^2+^ concentration in the supernatant was also clearly decreased upon treatment with AADAAC-F4. Based on this result, it was revealed that AADAAC-F4 could capture not only Cd^2+^ but also Zn^2+^. However, the removal rate upon the AADAAC-F4 treatment for Zn^2+^ was slightly lower than that of Cd^2+^ (69.9% for Zn^2+^ and 85.5% for Cd^2+^, respectively). This was consistent with the observation that the AADAAC sequence preferentially binds Cd^2+^ over Zn^2+^ at pH 8.0^53^. Since this sequence binds to Zn^2+^ more strongly than to Cd^2+^ above pH 11.0, we suggest that the AADAAC-conjugated ELPs can be used as metal scavengers for different metal ions by changing the pH of the solution.

**Figure 4:**
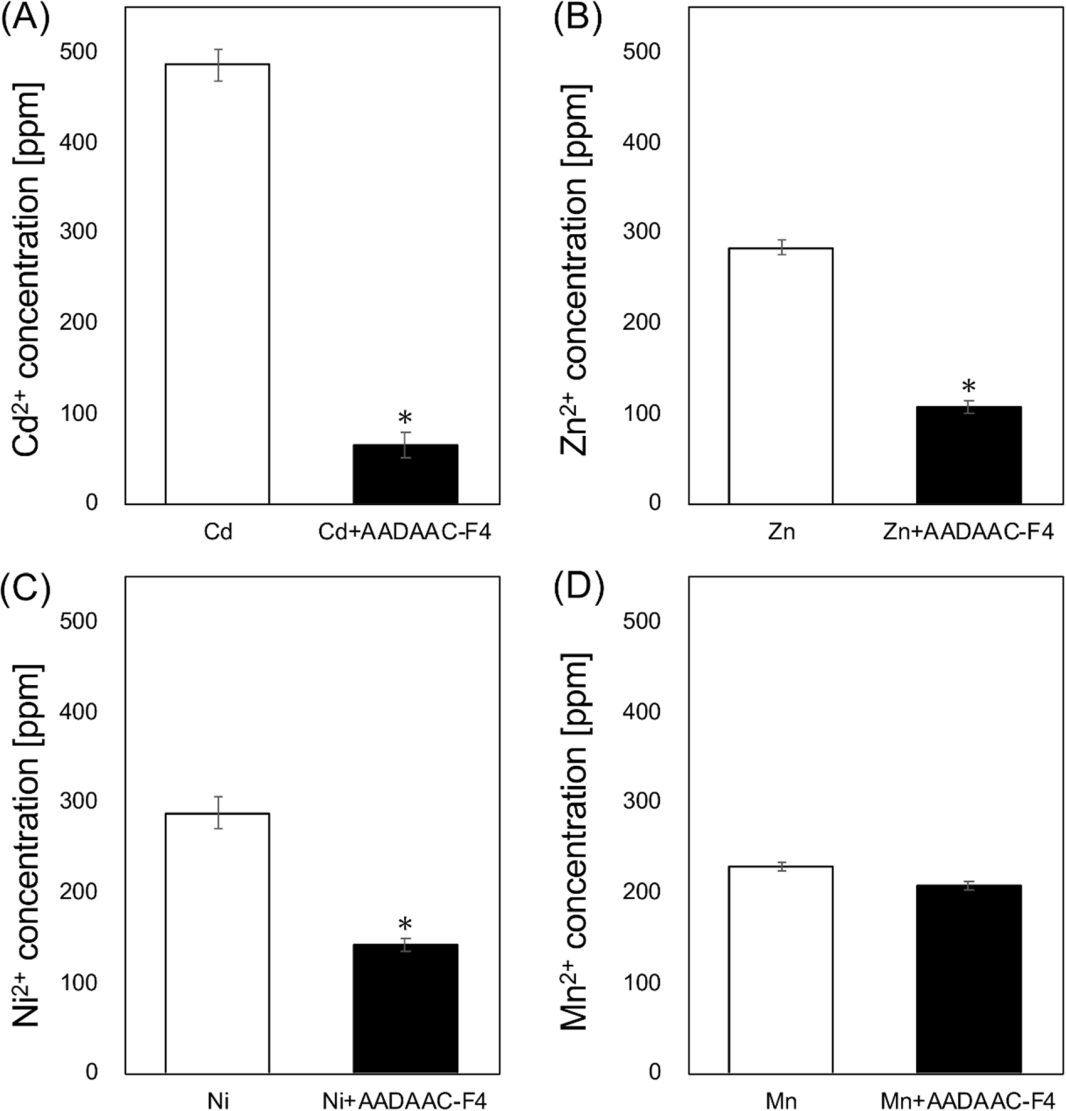
Metal binding affinity of AADAAC-F4 analogues. AADAAC-F4 was dissolved in Tris–HCl buffer solution (50.0 mM Tris, 599 mM NaCl, pH 8.0) at a concentration of 10 mg/mL (4.26 mM) and equimolar amounts of (**A**) Cd^2+^, (**B**) Zn^2+^, (**C**) Ni^2+^, and (**D**) Mn^2+^, dissolved in 1 M HNO_3_, were added. After incubation at 50 °C, the concentration of each metal ion in the supernatant was measured by ICP-MS. *P < 0.05 in a *t*-test between the concentration after treatment with each peptide compared to that of the control in each graph.

To evaluate the potential of AADAAC-F4 as a scavenger for metal ions other than Cd^2+^ and Zn^2+^, the metal binding properties of aqueous solutions of NiCl_2_ and MnSO_4_, in addition to CdCl_2_ and ZnCl_2_, were analyzed by ICP-MS in the presence or absence of the peptide. Equal molar amounts of peptides and metal ions were dissolved in the solution. After treatment with AADAAC-F4, quantitative analysis was carried out using ICP-MS to evaluate the concentration of each metal ion in the supernatant liquid phase. As shown in Fig. 4, the concentrations of Cd^2+^ and Zn^2+^ decreased by treatment with AADAAC-F4 (the removal rate was 86.7% and 62.3%, respectively) (Table 4). These results were consistent with the colorimetric study described above. It was previously reported that the AADAAC sequence also forms a complex with Ni^2+^^53^. However, the decrease in Ni^2+^ concentration in this measurement was slightly lower than that of Cd^2+^ and Zn^2+^ concentrations (the removal rate of Ni^2+^ was 50.6%). It was also revealed that AADAAC-F4 showed little affinity for Mn^2+^; the removal rate was 9.4% and no significant difference was found with the control group. The binding affinity of metal ions to AADAAC-F4 was qualitatively consistent with the hard-soft acid–base theory; the binding affinity of soft metals to the peptides was relatively higher than that of hard metals. The metal-binding site of the AADAAC sequence consists of the terminal amino group, the internal aspartyl carboxylate, and the cysteinyl thiolate group^53^. For Ni^2+^, the amino terminus (hard base) is the primary ligating site, and the aspartyl residue can enhance the thermodynamic stability of Ni^2+^ complexes. On the other hand, the thiolate function (soft base) is the primary binding site for Zn^2+^ and especially Cd^2+^ ions. Although both amide nitrogen and thiolate sulfur atoms are involved in metal binding, the coordination of the thiolate group with hard metal ions is only desirable at high pH values. Actually, the formation of the AADAAC sequence-Ni^2+^ complex can compete with that of the complex with Cd^2+^ at pH values higher than 11.0^53^. In addition, it was also indicated that Na^+^ ions (599 mM), which were present in high concentrations in the solution, seem to have little effect on the binding of peptides to soft metal ions. Hence, it was suggested that AADAAC-F4 can be utilized as a superior scavenger for soft metal ions such as Cd^2+^ in neutral and weakly basic aqueous solutions. Moreover, the fact that the presence of sodium ions does not affect the binding of heavy metal ions to AADAAC-F4 is considered to be a great advantage in using this peptide as a metal scavenger in the environment.

**Table 4:** The removal rate of various metal ions with the peptide treatment determined by ICP-MS measurement.

Metal ions	Concentration of the metal ions (ppm)		Removal rate (%)
	Control	+ AADAAC-F4	
Cd^2+^	485.9 ± 17.6	64.4 ± 13.6	86.7 ± 0.03
Zn^2+^	283.3 ± 8.6	106.8 ± 7.4	62.3 ± 0.03
Ni^2+^	287.4 ± 18.0	141.9 ± 7.0	50.6 ± 0.02
Mn^2+^	228.1 ± 5.4	206.7 ± 4.8	9.4 ± 0.02

Mean removal rates with SE are shown in the table. Equal molar amounts of peptides and metal ions were dissolved in Tris–HCl buffer solution (50.0 mM Tris, 599 mM NaCl, pH 8.0). The measurements were repeated at least three times.

### Recycling of AADAAC-F4 and re-evaluation of its Cd^2+^ binding affinity

**Figure 5:**
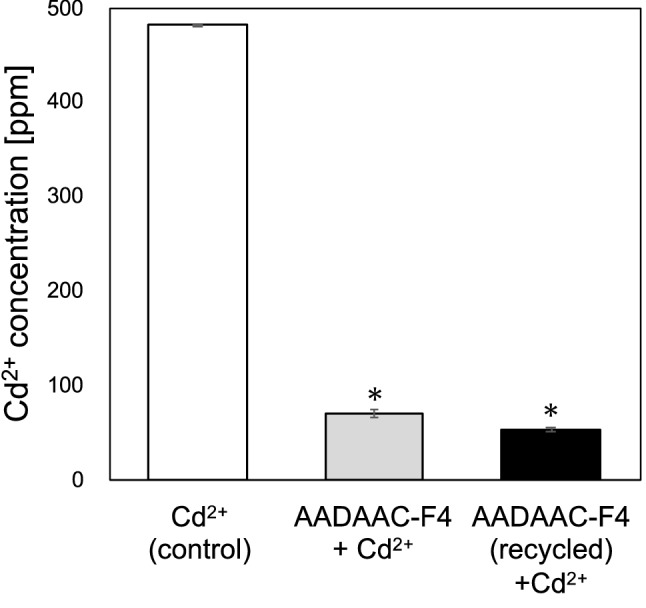
Cd^2+^ binding affinity of regenerated AADAAC-F4. Concentration of Cd^2+^ in the supernatant of the CdCl_2_ solution (4.26 mM) measured after treatment with AADAAC-F4 or recycled AADAAC-F4. *P < 0.05 in a *t*-test between the concentration after treatment with each peptide compared to that of the control. There was no significant difference in Cd^2+^ concentration between AADAAC-F4 + Cd^2+^ and AADAAC-F4 (recycled) + Cd^2+^.

To evaluate the feasibility of recycling and reusing AADAAC-F4 as metal scavengers, the post-use recovery conditions and the Cd^2+^ binding affinity of the recycled peptide were investigated. A previous study indicated that the metal-binding capacity of the AADAAC peptide is reduced in acidic solution^53^. Therefore, it was presumed that dissolving Cd^2+^-bound AADAAC-F4 in acidic solution would release Cd^2+^ from the peptide and the Cd^2+^ free peptide could then be reused as metal scavenger. To confirm the validity of this presumption, we examined whether the Cd^2+^-free AADAAC-F4 peptides could be obtained by treating Cd^2+^-bound-AADAAC-F4 with 1 M hydrochloric acid solution. Since there was a possibility that the AADAAC-F4 could dimerize during the regeneration process, a UPLC-MS measurement was also performed after the regeneration treatment (Fig. S5). We observed that the dimerized peptide was not detected after the treatment. After the recycling of AADAAC-F4, colorimetric analysis was performed to confirm whether the recycled AADAAC-F4 retained its Cd^2+^ binding capacity. We observed that the Cd^2+^ concentration in the supernatant was reduced to 11.0% (the removal rate was 89.0%) by the regenerated AADAAC-F4 (Fig. 5 and Table 5). There was no significant difference in Cd^2+^ removal rates between AADAAC-F4 (first use) and recycled AADAAC-F4. Thus, we confirmed that AADAAC-F4 was successfully recycled by this optimized recycle method using simple treatment with the acidic solution. The observation that the peptides can be recycled indicates that economical metal recovery can be achieved using the AADAAC-F4.

**Table 5:** The removal rate of Cd^2+^ with the recycled AADAAC-F4 treatment.

	Concentration of peptides		Concentration of metal ion	Removal rate
	mg/mL	mM	ppm	%
AADAAC-F4: Cd^2+^ (ratio = 1:1)	0	0	481.7 ± 0.024	89.0 ± 0.005
	10	4.26	53.0 ± 2.3	

Cd^2+^ bound-AADAAC-F4 was treated with 1 M of hydrochloric acid solution and then evaluated its metal binding affinity. Mean removal rate with SE were shown in the table. The peptide was dissolved in Tris–HCl buffer solution (50.0 mM Tris, 599 mM NaCl, pH 8.0). The measurements were repeated at least three times.

### Size distribution of the coacervates of AADAAC-F4

**Figure 6:**
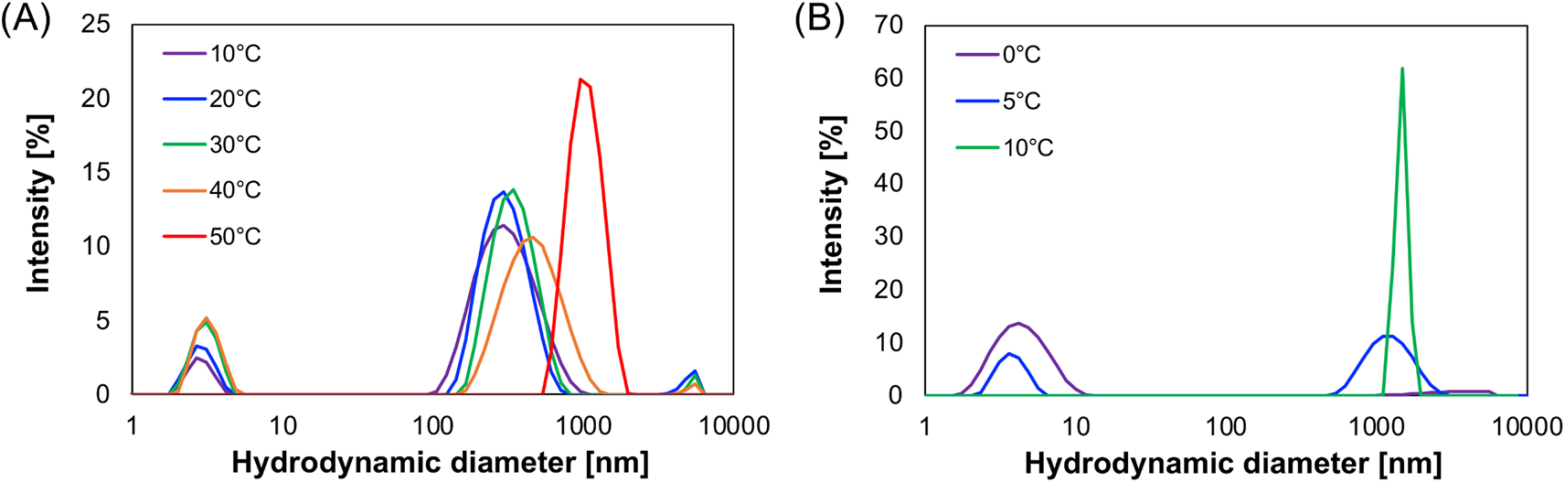
Particle size distribution of AADAAC-F4 at various temperatures. The size distribution of the coacervates formed in the AADAAC-F4 aqueous solution were analyzed by DLS. (**A**) AADAAC-F4 was dissolved in Tris–HCl buffer solution (50.0 mM Tris, 599 mM NaCl, pH 8.0). The peptide concentration was 4.26 mM. Under this condition, the *T*_t_ value for AADAAC-F4 was 45.2 °C. (**B**) AADAAC-F4 was dissolved in the same Tris–HCl buffer containing equimolar amount of CdCl_2_. Under this condition, the *T*_t_ value for AADAAC-F4 was 2.2 °C.

The formation of coacervates accompanying the increase in temperature of the AADAAC-F4 solutions was investigated by measuring their size distribution by dynamic light scattering (DLS) analysis at 10–50 °C (Fig. 6A). For this analysis, AADAAC-F4 was dissolved in a Tris–HCl solution under the same conditions used for turbidity measurements. The hydrodynamic diameter distribution of AADAAC-F4 was approximately 200 nm between 10 and 40 °C. Although there was no apparent increase in turbidity in this temperature range, AADAAC-F4 formed sub-micron aggregates under *T*_t_. When the solution temperature increased to 50 °C, a larger hydrodynamic diameter (approximately 1–2 µm) for the particles was observed. These results indicated that the sub-micron aggregates matured into micrometer-sized aggregates at temperatures above *T*_t_. The particle size measurement analyses indicated that the coacervation of AADAAC-F4 might follow a stepwise process in which sub-micron aggregates grow into micrometer-sized coacervates. Such a stepwise process is similar to that of the dendritic thermoresponsive ELP^55^ and some strong coacervatable short ELPs that we reported earlier^49–51^. In addition, DLS measurements were carried out in the presence of Cd^2+^ (Fig. 6B). Although the particle size of AADAAC-F4 was 3–4 nm at 0 °C, the particles immediately matured to micrometer-sized coacervates at 5–10 °C. When the solution temperature was raised to 15 °C, the particles precipitated and the particle size could not be measured. Thus, it was considered that the self-assembly ability of AADAAC-F4 is significantly enhanced in the presence of Cd^2+^. This result is consistent with the result from turbidity measurement described above.

### Morphology of peptide aggregates

**Figure 7:**
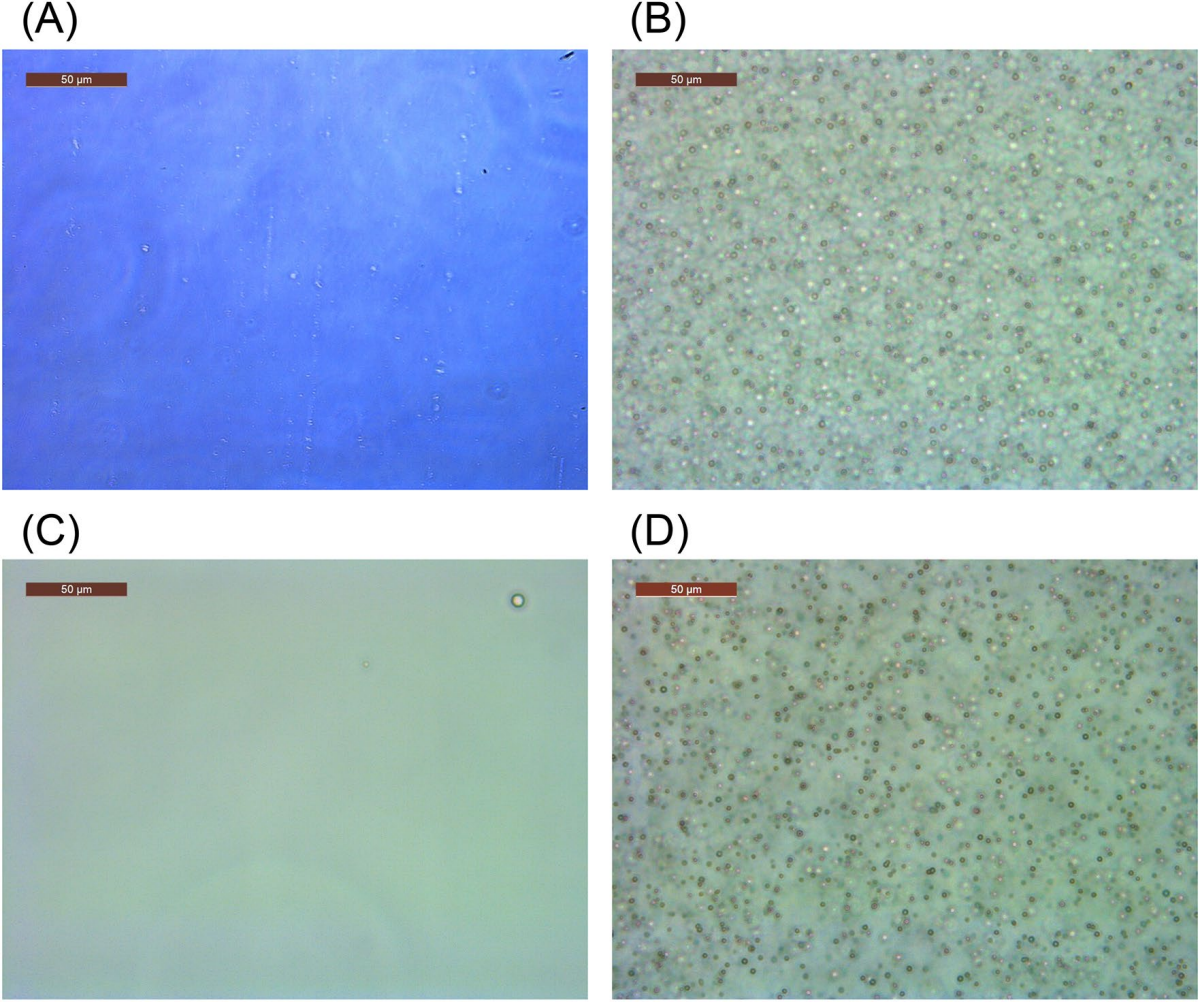
Optical microscopy images of AADAAC-F4 in solution. Images of AADAAC-F4 solution (4.26 mM) dissolved in Tris–HCl buffer (50.0 mM Tris, 599 mM NaCl, pH 8.0) obtained at (**A**) 10 °C, (**B**) 40 °C, and (**C**) 20 °C (after cooling from 40 °C). (**D**) AADAAC-F4 solution (4.26 mM) dissolved in Tris–HCl buffer in the presence of equimolar amounts of Cd^2+^ at 4 °C. Scale bar = 50 μm.

**Figure 8:**
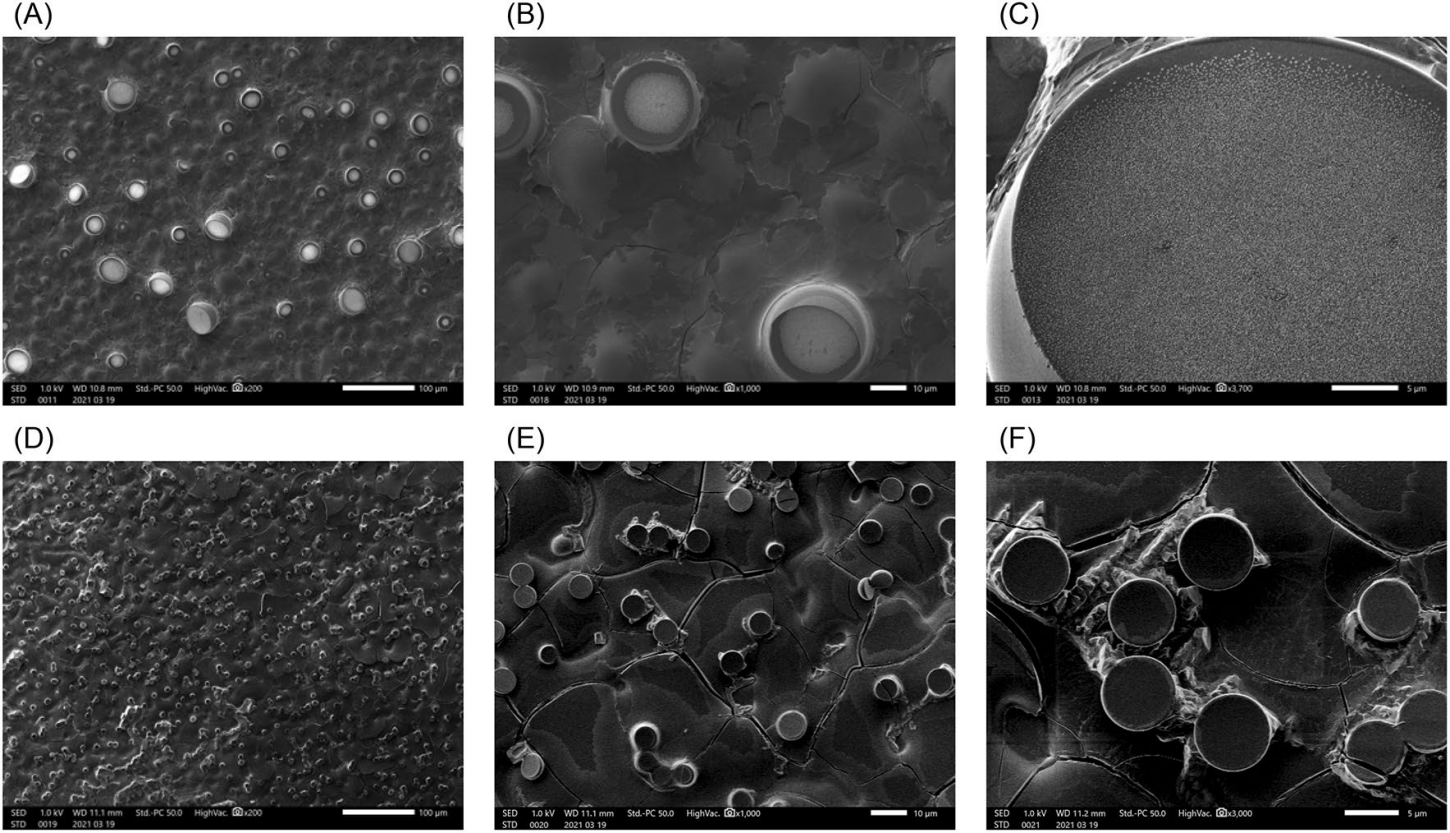
SEM image of self-assembled structure of AADAAC-F4 coacervated at 25 °C. (**A**–**C**) SEM images of the coacervate of AADAAC-F4 in the absence of Cd^2+^. The magnifications for the images are 200 × for (**A**), 1000 × for (**B**), and 3700 × for (**C**). (**D**–**F**) SEM images of the coacervate of AADAAC-F4 in the presence of Cd^2+^. The magnifications for the images are 200 × for (**D**), 1000 × for (**E**), and 3000 × for (**F**).

The aggregates of ELP analogues were observed using optical microscopy to obtain relevant morphological information. The observed aggregates of AADAAC-F4 in Tris–HCl buffer are shown in Fig. 7. When a homogeneous peptide solution with free Cd^2+^ (20 °C, Fig. 7A) was heated to 40 °C, spherical aggregates of AADAAC-F4 with a diameter of approximately 3 µm were observed (Fig. 7B). Subsequently, when the solution was cooled to 20 °C, the aggregates returned to their original solubilized state (Fig. 7C). On the other hand, similar AADAAC-F4 aggregates were observed at 4 °C in the presence of Cd^2+^ (Fig. 7D). These results demonstrate that the appearance/disappearance of spherical aggregates of the peptides is temperature dependent, which is consistent with the results obtained from turbidity measurements. Additional morphological analysis using scanning electron microscopy (SEM) was carried out to obtain structural information on the submicron coacervates of AADAAC-F4 (Fig. 8). Peptide samples prepared from the solution of AADAAC-F4 in Tris–HCl buffer without Cd^2+^ were initially analyzed; spherical particles with a diameter of 5–20 μm, formed by aggregation of AADAAC-F4, were clearly observed (Fig. 8A). We further found that many of the spherical particles had been ruptured during the vacuum process. In addition, these peptide particles contained a large number of small square crystals inside the particles, which might have been formed due to the added NaCl (Fig. 8B,C). It was considered that these large particles were formed over time with the concentration of the peptide solution increasing during air drying. It was also presumed that NaCl crystals were produced simultaneously during the drying process. On the other hand, when the AADAAC solution containing Cd^2+^ was used for sample preparation, a number of small particles (up to 5 µm in diameter) were observed (Fig. 8D). Small salt crystals were also observed inside these small aggregates (Fig. 8E,F). These SEM observations showed that the different self-assembling process of AADAAC-F4 was significantly enhanced in the presence or absence of Cd^2+^ ions. In the presence of Cd^2+^, the hydrophobicity of AADAAC-F4 was improved by binding with Cd^2+^ as described above. Thus, small coacervates were formed simultaneously, which then precipitated at about the same time. In contrast, in the absence of Cd^2+^, the AADAAC-F4 showed only weak self-association ability. Therefore, the peptide aggregates were considered to have formed when the solution was concentrated and then matured by a stepwise agglutination process that was revealed by the DLS measurements. AADAAC-F4 can rapidly form spherical peptide particles of several micrometers in diameter, indicating its advantageous easy separation from the solution when used as a recyclable metal ion sequestering agent.

### Binding thermodynamics of the metal/AADAAC-F4 interaction

**Figure 9:**
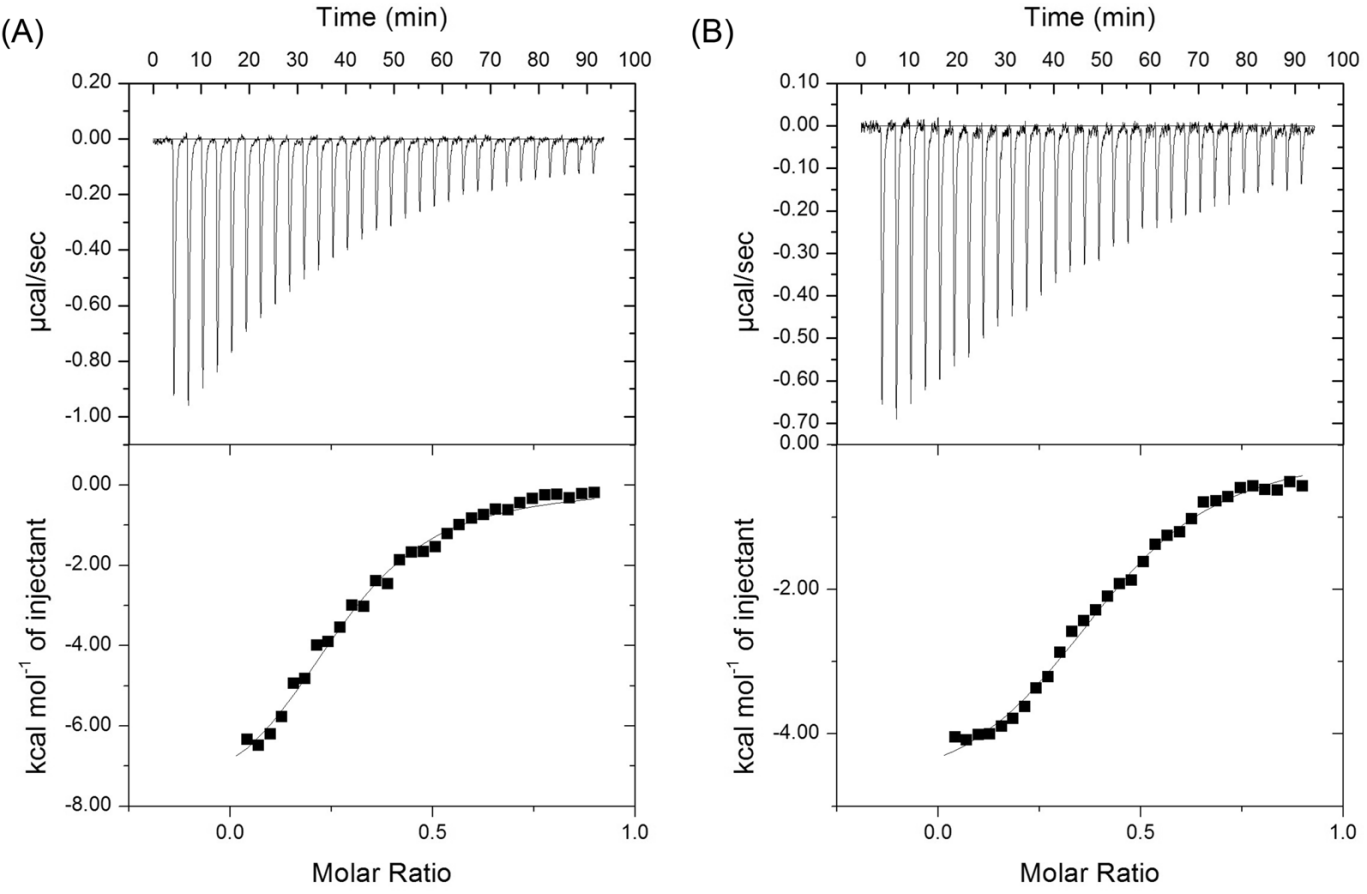
ITC curves obtained with the titration of (**A**) 1.0 mM CdCl_2_ or (**B**) 1.0 mM ZnCl_2_ to 0.1 mM AADAAC-F4 at 30 °C in Tris buffer at pH 8.0. (**A**) Fit line for Cd: *n* = 0.263 ± 0.016, *K*_a_ = 1.38 (± 0.81) × 10^5^, Δ*H* =  − 10.84 ± 1.03 kcal mol^−1^, and Δ*S* =  − 0.012 ± 0.003 kcal K^−1^ mol^−1^. (**B**) Fit line for Zn: *n* = 0.354 ± 0.046, *K*_a_ = 1.24 (± 0.71) × 10^5^, Δ*H* =  − 6.82 ± 0.81 kcal mol^−1^, and Δ*S* = 0.0004 ± 0.003 kcal K^−1^ mol^−1^. The metal-dependent thermodynamics are summarized in Table 6.

Isothermal titration calorimetry was performed to investigate the binding dynamics of metal ions to AADAAC-F4 (Fig. 9). To eliminate the influence of peptide aggregation, these measurements were performed using a low-concentration of peptide solution (0.1 mM). The free energy and entropy of Cd^2+^ or Zn^2+^ binding to AADAAC-F4 were determined from the obtained parameters (enthalpy, binding affinity, and stoichiometry) (Table 6). When the contribution of the enthalpy and entropy to the binding free energy was examined, it was found that metal binding to AADAAC-F4 was predominantly enthalpically driven. In this assay condition, the binding affinity of Cd^2+^ to AADAAC-F4 was only slightly stronger than that of Zn^2+^. On the other hand, the Δ*H* of Cd^2+^ binding was significantly larger than that of Zn^2+^ binding. This result was consistent with the previous result showing that the thermodynamic stability of the complex formed by Zn^2+^ and the AADAAC sequence was significantly lower than that of the complex formed by Cd^2+^ and the AADAAC sequence^53^. The stoichiometry values *n* were 0.263 for Cd^2+^ and 0.354 for Zn^2+^. These small *n* values (n < 1) indicated that multiple peptides coordinated with one metal atom; in other words, the result of ITC measurements suggested that metal-to-peptide stoichiometry was different from 1:1. In addition, UPLC-MS analysis also suggested that a complex consisting of three molecules of AADAAC-F4 and one Cd^2+^ ion would be formed (Fig. S6). Nevertheless, colorimetric analysis and ICP-MS measurements using equimolar amounts of peptides and metals showed a high cadmium removal rate of ~ 85%. This result suggested that most of the removed metal ions were incorporated into the aggregates formed by the peptides and precipitated. In the turbidity measurement described above, it was shown that the binding of the metal ions to AADAAC-F4 greatly increased the hydrophobicity of this peptide and significantly enhanced its self-association ability. Cadmium ion and, to a lesser extent, zinc ion formed thermodynamically more stable complexes with AADAAC-F4 than the other metals, suggesting that Cd^2+^ and Zn^2+^ are more effective than the other metal ions in enhancing peptide aggregation. This could be the cause behind the higher removal rate of Cd^2+^ in relation to that of Zn^2+^. Therefore, it was considered that the formation of thermodynamically stable complexes between AADAAC-F4 and metal ions is important for increasing the removal rate of the metal ions.

**Table 6:** Thermodynamic parameters for Cd^2+^ or Zn^2+^ titration into the AADAAC-F4 solution.

Titrant	*n*	*K*_a_ (M^−1^)	Δ*G *(kcal mol^−1^)	Δ*H *(kcal mol^−1^)	TΔ*S *(kcal mol^−1^)
CdCl_2_	0.263	1.38 × 10^5^	− 7.11	− 10.84	− 3.73
ZnCl_2_	0.354	1.24 × 10^5^	− 6.95	− 6.82	0.12

Isothermal titration calorimetry was performed at 30 °C in 50.0 mM Tris buffer containing 599 mM of NaCl at pH 8.0.

## Conclusion

In this study, novel ELP analogues were synthesized to develop metal-binding ELPs as easy-to-use metal ion scavengers. Turbidity measurements of AADAAC-F4 demonstrated that this peptide exhibited coacervation ability similar to that observed for the original (FPGVG)_n_ analogues. The self-assembly ability of AADAAC-F4 was significantly enhanced in the Cd^2+^-containing solution. The investigation of metal-binding property revealed that AADAAC-F4 molecules could capture soft metal ions, such as Cd^2+^ or Zn^2+^, with high binding affinity in the presence of high concentrations of Na^+^. From the experiments using the AADAAC-F4-dimer, it was revealed that the free thiol group of the AADAAC sequence is required for the specific metal binding activity and the associated lowering of the aggregation temperature. In addition, AADAAC-F4 can be readily recycled by simple treatment with an acidic solution and can be reused to collect Cd^2+^. Particle size distribution analysis indicated that AADAAC-F4 follows a stepwise self-association process involving the generation of sub-micron aggregates followed by coacervate maturation, which is similar to that observed for strong coacervatable short ELPs that we have previously reported^49–51^. Furthermore, microscopic analyses of AADAAC-F4 suggested that this peptide rapidly forms spherical particles several micrometers in diameter in the presence of Cd^2+^, implying that it can be easily separated from solution when used as a metal scavenging agent. From ITC measurements, it was suggested that the formation of thermodynamically stable complexes may be important for increasing the removal rate of the metal ions. Our analyses suggest that metal-binding short ELPs can be developed for various metal ions by introducing corresponding peptide sequences or chelating molecules with a variety of metal ion binding potencies. Although AADAAC-F4 can be synthesized chemically as a 26-mer peptide, further development of shorter, easier-to-synthesize, and more economical analogues will be necessary to facilitate the widespread use of peptide metal recovery agents. In conclusion, we demonstrated that thermoresponsive biomaterials with metal-binding capabilities have the potential to be used as versatile materials for the removal of metal ions from a variety of wastes.

## Materials and methods

### Chemicals

Fmoc-amino acids were purchased from Merck Ltd. (Darmstadt, Germany). Fmoc-NH-SAL-MBHA resin (100–200 mesh); *N,N*-diisopropylethylamine (DIPEA); and trifluoroacetic acid (TFA) were purchased from Watanabe Chemical Industries Ltd. (Hiroshima, Japan). 2-(1H-benzotriazole-1-yl)-1,1,3,3-tetramethyl uronium hexafluorophosphate (HBTU) and 1-hydroxybenzotriazole (HOBt) were purchased from Kokusan Chemical Co., Ltd. (Tokyo, Japan). Triisopropylsilane (TIS) and xylenol orange were purchased from Tokyo Chemical Industry Co., Ltd. (Tokyo, Japan). Hydrochloric acid (HCl); nitric acid; 1,2-ethanedithiol (EDT); CdCl_2_·2.5H_2_O; NiCl_2_·6H_2_O; MnSO_4_·5H_2_O; and tris(hydroxymethyl)aminomethane (Tris) were purchased from Nacalai Tesque Co. Ltd (Kyoto, Japan). ZnCl_2_ and Multielement Standard Solution W-V were purchased from FUJIFILM Wako Pure Chemical Corporation (Osaka, Japan). Water for the experiments was purified by Milli-Q Integral 3 (Merck Millipore, Darmstadt, Germany). Other solvents and reagents were obtained from commercial suppliers and used without further purification.

### Synthesis of elastin-like peptides

Elastin-like peptide analogues, H-(FPGVG)_n_-NH_2_ (n = 4 or 5, abbreviated as F4 or F5, respectively) and H-AADAAC-(FPGVG)_4_-NH_2_ were synthesized by an ABI 433A peptide synthesizer (Applied Biosystems, Foster city, CA, USA) using the solid-phase method with Fmoc chemistry, as described in our previous studies^49, 50^. For the peptide synthesis, HBTU (0.45 M) and HOBt (0.45 M) in *N*,*N*-dimethylformamide (DMF) were used as condensing agents in the FastMoc 0.25 mmol program included in SynthAssist 2.0 software (Applied Biosystems). After peptide chain elongation, the peptides, except for AADAAC-F4, were cleaved from the resin with a reagent cocktail containing 95% TFA/2.5% TIS/2.5% H_2_O. AADAAC-F4 was cleaved from the resin with a reagent cocktail containing 94% TFA/2.5% EDT/2.5% H_2_O/1.0% TIS. After cleavage, the resulting mixture was poured into 50 mL of diethyl ether and centrifuged to separate peptide precipitates from the cocktail. The crude peptide analogues were then pre-purified using a Sep-Pak Vac 35 cc C18 cartridge (Waters Co., Milford, MA) before final purification by HPLC. All peptides were dissolved in 15% acetonitrile aqueous solution, applied to the Sep-Pak cartridge, and an acetonitrile aqueous solution was poured into the Sep-Pak cartridge. The eluent solution was fractionated every 50 mL. The concentration of acetonitrile in the eluent was gradually increased to 15%, 30%, 40%, 60%, and 99% for peptide separation. Then, the eluent fraction containing the peptide was identified by UPLC-MS detection. The fraction was evaporated and lyophilized to obtain the peptide powder. Subsequently, further purification was performed by RP-HPLC (The Breeze 2 HPLC System, Waters Co.) using a C8 column (COSMOSIL 5C8-AR-300 Packed Column, 20 mm I.D. × 150 mm, C8-AP 5 μm, 300 Å, Nacalai Tesque Inc.) and a solvent system consisting of 0.1% TFA aqueous solution (v/v, solvent A) and a mixture of 80% acetonitrile and 20% solvent A (v/v, solvent B). The purified fraction was evaporated and lyophilized to obtain the desired peptide analogues. Purity and molecular weight of the peptides were confirmed by ACQUITY UPLC H-Class (Waters Co.) equipped with an ACQUITY UPLC BEH C-18 column (100 mm, flow rate 0.6 mL/min) (Waters Co.) at 49 °C, and the eluting product was detected by UV absorption at 225 nm using a quadrupole mass spectrometer, ACQUITY QDa (Waters Co.). The solvent system for UPLC consisted of a 0.1% formic acid aqueous solution (v/v, solvent A) and 0.1% formic acid in acetonitrile (v/v, solvent B); elution was performed with a linear gradient (24% to 56%) of solvent B over 4.23 min.

### Synthesis of AADAAC-F4-dimer by aerobic oxidation

AADAAC-F4-dimer was synthesized by aerobic oxidation of the AADAAC-monomer^47^. A total of 50.64 mg (21.5 µmol) of AADAAC-F4 was dissolved in 50 mL of a mixed solvent of acetonitrile and water (60:40, v/v). Then, 10 mg of ammonium bicarbonate was added and the resulting solution was stirred at 25 °C in open air. After completion of the reaction, the mixture was concentrated in vacuo. The residual crude product was purified by Sep-Pak cartridge filtration and analyzed by HPLC as described above.

### Turbidity measurement

The temperature-dependent self-assembling property of the ELP analogues was evaluated using a JASCO V-660 spectral photometer (JASCO Co.) as previously described^49, 50^. The peptide sample solutions were diluted to a concentration of 5.0 or 10 mg/mL with Tris–HCl buffer solution (50.0 mM Tris, 599 mM NaCl, at pH 8.0 or 50.0 mM Tris, 599 mM NaCl, 4.26 mM CdCl_2_ or ZnCl_2_, at pH 8.0). Turbidity was measured at 400 nm with increasing or decreasing temperature at a rate of 0.5 °C/min from 5 °C. Each concentration of sample solution was measured at least three times. The self-assembling property was described by the phase transition temperature (*T*_t_), which is the temperature at which the turbidity of the solution reaches half the maximum value.

### Spectrophotometric determination of the affinity of metal ions to AADAAC-F4

The colorimetric analysis of Cd^2+^ and Zn^2+^ was carried out to evaluate the amount of metal ions absorbed into the coacervation phase of the peptides by using spectral photometer JASCO V-660^50^. A concentration of 10 mg/mL (4.26 mM) of AADAAC-F4 sample solutions in Tris–HCl buffer solution (50.0 mM Tris, 599 mM NaCl, pH 8.0) containing 4.26 mM CdCl_2_, 3.19 mM CdCl_2_ or 4.26 mM ZnCl_2_ were prepared. The peptide solution was incubated at 4 °C overnight and then at 40 °C for an hour to separate the lower coacervation phase from the upper equilibrium solution phase. After incubation, the peptide solution was immediately centrifuged at room temperature for 2 min (6,200 rpm) to remove the aggregates. An aliquot of 50 µL of supernatant of the equilibrium solution phase and 1 M xylenol orange (XO) aqueous solution were added to 900 µL of acetate buffer solution and incubated for 10 min. Subsequently, the concentration of Cd^2+^ was determined by measuring the absorbance at 575 nm corresponding to the absorbance of the cadmium-XO complex. The concentration of Zn^2+^ was also determined by measuring the absorbance at 550 nm corresponding to the absorbance of the zinc-XO complex in the same manner. The amount of metal ion absorbed in the peptide solution was determined by the calibration line, which was prepared in the same manner by using a series of standard CdCl_2_ aqueous solutions of known concentrations (0–5.0 ppm) and standard ZnCl_2_ aqueous solutions of known concentrations (0–2.0 ppm). The homogeneity of variances between the concentration of each metal ion in the supernatant and control solution was confirmed by the F test. The statistical difference between the concentration of each metal ion in the supernatant and control solution was determined by the Student's *t* test. Results were considered statistically significant at *P* values ≤ 0.05.

### Determination of the affinity of various metal ions to AADAAC-F4 by ICP-MS

Quantitative analysis of the affinity of Cd^2+^, Zn^2+^, Ni^2+^, and Mn^2+^ to AADAAC-F4 was carried out by ICP-MS to analyze the absorption of the metal ions into the coacervation phase of AADAAC-F4^50^. The quantitative analysis was performed using an Agilent Technologies 7500c ICP-MS system (Agilent Technologies, Inc., Santa Clara, CA). AADAAC-F4 was dissolved in 99 µL of Tris–HCl buffer solution (50.0 mM Tris, 599 mM NaCl, pH 8.0) at a concentration of 10 mg/mL (4.26 mM). Then, 1 µL of the metal salt solution (426 mM) was added to 1 M HNO_3_ aqueous solution. The resulting solution was incubated overnight at 4 °C and then at 50 °C for an hour to separate the lower coacervation phase from the upper equilibrium solution phase. After incubation, the peptide solution was immediately centrifuged at 25 °C for 2 min (14,000 rpm) to remove aggregates. Then, 6.7 μL of the supernatant of the equilibrium solution phase was diluted to 10 mL with 10 mM HNO_3_ aqueous solution. The resulting solution was filtered using a syringe filter (pore size, 0.45 μm) (Sartorius, Goettingen, Germany). The concentration of each metal ion in the resulting solution was analyzed using ICP-MS. The amount of metal ion absorbed in the peptide solution was determined by the calibration line prepared using Multielement Standard Solution W–V. Each measurement was performed at least three times. The statistical analyses were carried out in the same manner as described above.

### Recycle of the AADAAC-F4

To examine the possibility of recycling AADAAC-F4, 26.73 mg of the peptide was dissolved in 3.0 mL of Tris buffer solution containing 4.26 mM CdCl_2_ at 4 °C overnight. The peptide solution was incubated at 40 °C for an hour. After incubation, the peptide solution was immediately centrifuged at room temperature for 2 min (6,200 rpm) to remove aggregates. The supernatant was removed and the resulting precipitates were redissolved in 3.0 mL of HCl (1 M). The pH of the resulting solution was adjusted to 2.2 by adding 1.0 mM of NaOH aqueous solution, for purification. Then, AADAAC-F4 was separated using Sep-Pak Vac 35 cc C18 cartridge and lyophilized as described above. To examine the metal-binding property of recycled AADAAC-F4, the colorimetric analysis of Cd^2+^ was performed again using the recycled peptide in the same manner as described above. The statistical analyses were carried out in the same manner as described above.

### Dynamic light scattering (DLS) analysis

The distribution of the particle size in the AADAAC-F4 solution was analyzed by DLS measurement using Zetasizer Nano ZS (Malvern Instruments Ltd., Worcestershire, UK) in a measurement cell (ZEN0112; Malvern Instruments Ltd.)^50^. The AADAAC-F4 aqueous solution was prepared at a concentration of 10 mg/mL (4.26 mM) in Tris–HCl buffer solution (50.0 mM Tris, 599 mM NaCl, pH 8.0). DLS analysis was performed by increasing the temperature at 10 °C intervals from 10 to 50 °C. In addition, similar measurements were also carried out in the presence of CdCl_2_ (4.26 mM) by increasing the temperature at 5 °C intervals from 0 to 15 °C. Measurement duration was selected automatically. Parameter dataset “Protein” (dataset: refractive index, 1.450; absorption, 0.001) was used as the material parameter, and parameter dataset “Water” (dataset: refractive index, 1.330; viscosity, 0.8872) was chosen as the dispersant parameter. Attenuation was selected automatically. The measurement of each concentration was performed at least three times.

### Microscopic study of AADAAC-F4

The morphology of the coacervates of AADAAC-F4 was observed by optical microscopy. The light field observation was performed using a Leica DM IL LED (Leica Microsystems CMS, Wetzlar, Germany) equipped with HI PLAN 40 × (Leica Microsystems CMS) and HC PLAN 10 × (Leica Microsystems CMS))^49, 50^. The peptide sample solutions were diluted to a concentration of 10 mg/mL with Tris–HCl buffer solution (50.0 mM Tris, 599 mM NaCl, at pH 8.0 or 50.0 mM Tris, 599 mM NaCl, 4.26 mM CdCl_2_, at pH 8.0), or phosphate buffer, and applied on a glass slide. Sample imaging was performed at various temperatures between 4 and 50 °C by using Thermo Plate TP-CHSQM (Tokai Hit Co., Ltd., Shizuoka, Japan).

### Scanning electron microscopy

An aqueous solution of 10 mg/mL of AADAAC-F4 (in Tris–HCl buffer as described above) was dropped onto a cover glass without or with Cd^2+^ (4.26 mM) and left at 25 °C for air drying. Subsequently, the residue was rinsed gently three times with distilled water, and air-dried on the cover glass surface. The prepared sample was platinum sputter coated (5 nm thick) and examined with a SU3500 microscope (Hitachi High-Tech Corporation, Tokyo, Japan) at an operating voltage of 5.00 kV^50^.

### Isothermal titration calorimetry

Calorimetric measurements were carried out on a VP-ITC MicroCal titration calorimeter (MicroCal, Inc., Northampton, MA, USA)^56^. AADAAC-F4, CdCl_2_, and ZnCl_2_ were dissolved in Tris–HCl buffer solution (50.0 mM Tris, 599 mM NaCl, pH 8.0). The peptide concentration in the cell was 0.1 mM and the metal concentration in the syringe was 1.0 mM. ITC measurements were carried out at 30 °C with 25–30 injections of 4 μL of titrant (with intervals of 180 s between injections) and stirring at 310 rpm. The heat of dilution, measured by the injection of titrant to the buffer solution, was subtracted from each titration to obtain the net reaction heat value. At least three independent measurements were performed for each metal ion and the best-fit values were averaged and reported. The data are presented as the baseline-adjusted raw data in the upper panel and the integrated heat values (from the upper panel) as the function of a metal-to-peptide ratio in the cell in the direct titration in the lower panel. The fitting of the experimental data to a theoretical titration curve by a nonlinear least square algorithm was carried out using MicroCal Origin software with Δ*H* (enthalpy change in kcal mol^−1^), *K*_a_ (association constant in M^−1^), and *n* (number of binding sites) as adjustable parameters. Thermodynamic parameters were calculated from the equation,

$$\Delta G = \, \Delta H{-}T\Delta S \, = \, {-}RT{\text{ln}}K_{a}$$

where Δ*G*, Δ*H*, and Δ*S* are the changes in free energy of Gibbs, enthalpy, and entropy of binding, respectively. *T* is the absolute temperature (303 K in this work) and the gas constant *R* = 1.98 cal mol^−1^ K^−1^.

## Supplementary Information

Supplementary Information.

## Data Availability

The datasets used during the current study are available from the corresponding author upon reasonable request.
