# Supplementary material for: Metal ion scavenging activity of elastin-like peptide analogues containing a cadmium ion binding sequence
Source: Sci Rep. 2022 Feb 3;12:1861. doi: 10.1038/s41598-022-05695-w (PMC8814041; doi:10.1038/s41598-022-05695-w)
Supplement: Supplementary file 1 — Supplementary Information. [file 41598_2022_5695_MOESM1_ESM.docx]

**Supplementary information**

**Metal ion scavenging activity of elastin-like peptide analogues containing a cadmium ion binding sequence**

AUTHOR NAMES
Shogo Sumiyoshi,^1^ Keitaro Suyama,^2^ Daiki Tatsubo,^1^ Naoki Tanaka,^1^ Keisuke Tomohara,^2^ Suguru Taniguchi,^3^ Iori Maeda,^3^ and Takeru Nose^1,2,*^

AUTHOR ADDRESS
^1^Laboratory of Biomolecular Chemistry, Department of Chemistry, Faculty and Graduate School of Science, Kyushu University, Fukuoka 819-0395, Japan.

^2^Laboratory of Biomolecular Chemistry, Faculty of Arts and Science, Kyushu University, Fukuoka 819-0395, Japan.

^3^Department of Physics and Information Technology, Kyushu Institute of Technology, Iizuka, Fukuoka 820-8502, Japan.

Manuscript Correspondence:

Prof. Takeru Nose

Tel: +81-92-802-6025

Fax: +81-92-802-6025

e-mail: nose@artsci.kyushu-u.ac.jp

**Supporting Figures ..............................................................................................(S4–S9)**

Figure S1. UPLC-MS analysis of the synthesized peptide analogues.

Figure S2. Turbidity profiles of AADAAC-F4 without NaCl in solution.

Figure S3. Turbidity profiles of F5 containing 3.5% NaCl in solution.

Figure S4. Metal binding affinity of AADAAC-F4 analogues.

Figure S5. UPLC-MS analysis of the recycled AADAAC-F4.

Figure S6. UPLC-MS analysis of the Cd^2+^-AADAAC-F4 complex.

**Supporting Table ......................................................................................................(S10)**

Table S1. The removal rate of Cd^2+^ with the peptide treatment.

**Experimental procedure**

**UPLC-MS analysis of the complex of Cd^2+^ and AADAAC-F4.**

The stoichiometry of Cd^2+^ and AADAAC-F4 was investigated by UPLC-MS (ESI) analysis. AADAAC-F4 was dissolved in 10.0 mM NH_4_HCO_3_ aqueous solution (pH 8.6) at a concentration of 2.0 mg/mL (0.852 mM). Then, 0.852 mM of CdCl_2_ solution (in 50.0 mM Tris, 599 mM NaCl, pH 8.0) was added. Resulting solution was analyzed by ACQUITY UPLC H-Class (Waters Co.) equipped with an ACQUITY UPLC BEH C-18 column (100 mm, flow rate 0.6 mL/min) (Waters Co.) at 49°C and the eluting product was detected by UV absorption at 225 nm and a quadrupole mass spectrometer, ACQUITY QDa (Waters Co.). The solvent system for UPLC consisted of 10.0 mM NH_4_HCO_3_ aqueous solution (pH 8.6, solvent A) and 0.1% formic acid in acetonitrile (v/v, solvent B), and elution was performed with a linear gradient of solvent B, 24% to 56% over 4.23 min.

Figure S1. UPLC-MS analysis of the synthesized peptide analogues.

(A) F5, (B) F4, (C) AADAAC-F4, and (D) AADAAC-F4-dimer. The subscript for each peak indicates the retention time of the UPLC analysis and the mass number detected. Insets in each chart show the result of mass spectroscopy at each peak.

**Figure S2. Turbidity profiles of AADAAC-F4 without NaCl in solution.**

Turbidity changes of AADAAC-F4 in 10 mM of Tris-HCl buffer (50 mM, pH 8.0) associated with heating (solid line) was shown. AADAAC-F4 did not show turbidity changes in the absence of NaCl even the solution temperature was elevated to 90°C.

**Figure S3. Turbidity profiles of F5 containing 3.5% NaCl in solution.**

Turbidity changes of F5 in 10 mM of Tris-HCl buffer (50 mM, pH 8.0, containing 3.5% of NaCl) associated with heating (solid line) and cooling (dashed line) were shown.

Figure S4. Metal binding affinity of AADAAC-F4 analogues.

(A) Concentration of Cd^2+^ in supernatant of CdCl_2_ solution (4.26 mM) was measured after treatment with 1.07 mM (2.5 mg/mL), 2.13 mM (5.0 mg/mL), and 4.26 mM (10 mg/mL) of AADAAC-F4. (B) Relationship between the molar ratio of AADAAC-F4:Cd^2+^ and the removal rate of CdCl_2_. (C) Concentration of Cd^2+^ in supernatant of CdCl_2_ solution (4.26 mM) was measured after treatment with 2.13 mM (10 mg/mL) and 4.26 mM (20 mg/mL) of AADAAC-F4-dimer. (D) Relationship between the molar ratio of AADAAC-F4-dimer:Cd^2+^ and the removal rate of CdCl_2_. *: *P* < 0.05 in *t*-test between the concentration after treatment with each peptide compared to that of control in each graph.

Figure S5. UPLC-MS analysis of the recycled AADAAC-F4.

The subscript for each peak indicates the retention time of the UPLC analysis and the mass number detected.

Figure S6. UPLC-MS analysis of the Cd^2+^-AADAAC-F4 complex.

AADAAC-F4 was dissolved in ammonium hydrogen carbonate buffer solution at a concentration of 2.0 mg/mL (0.852 mM). Then, 0.852 mM of CdCl_2_ solution (in 50.0 mM Tris, 599 mM NaCl, pH 8.0) was added. Resulting solution was analyzed by UPLC-MS. (A) Analysis result of mass number in the range of 1170–1240. (B) Enlarged view of the range shown by the blue frame in the figure of (A). (C) Enlarged view of the range shown by the red frame in the figure of (A).

**Table S1. The removal rate of Cd^2+^** **with the peptide treatment.**

|  | Concentration of peptides | | Concentration of metal ion | | | Removal rate | | |
| --- | --- | --- | --- | --- | --- | --- | --- | --- |
|  | mg/mL | mM | ppm | | | % | | |
| AADAAC-F4: Cd^2+^  (ratio = 1:4) | 0 | 0 | 482.2 | ± | 4.4 | 17.9 | ± | 0.01 |
|  | 2.5 | 1.07 | 394.9 | ± | 5.4 |  |  |  |
| AADAAC-F4: Cd^2+^  (ratio = 1:2) | 0 | 0 | 482.2 | ± | 4.4 | 44.1 | ± | 0.01 |
|  | 5.0 | 2.13 | 269.7 | ± | 2.9 |  |  |  |
| AADAAC-F4: Cd^2+^  (ratio = 1:1) | 0 | 0 | 490.5 | ± | 2.6 | 85.5 | ± | 0.01 |
|  | 10 | 4.26 | 69.7 | ± | 3.4 |  |  |  |
| AADAAC-F4-dimer: Cd^2+^  (ratio = 1:2) | 0 | 0 | 477.9 | ± | 5.6 | 20.1 | ± | 0.03 |
|  | 10 | 2.13 | 382.0 | ± | 14.3 |  |  |  |
| AADAAC-F4-dimer: Cd^2+^  (ratio = 1:1) | 0 | 0 | 482.2 | ± | 4.4 | 31.5 | ± | 0.004 |
|  | 20 | 4.26 | 330.2 | ± | 1.9 |  |  |  |

Mean removal rate with SE was shown in the table. Each peptide was dissolved in Tris-HCl buffer solution (50.0 mM Tris, 599 mM NaCl, pH 8.0). The measurements were repeated at least three times.
